# Splenic abscesses as a first manifestation of Crohn’s disease: a case report

**DOI:** 10.1186/s12876-019-1066-1

**Published:** 2019-08-15

**Authors:** D. F. Bavaro, G. Ingravallo, F. Signorile, F. Fortarezza, F. Di Gennaro, G. Angarano, A. Saracino

**Affiliations:** 1 Department of Biomedical Sciences and Human Oncology, University of Bari “Aldo Moro”, Clinic of Infectious Diseases, Piazza G. Cesare, 11 - 70124 Bari, Italy; 2 Interdisciplinary Department of Medicine, University of Bari “Aldo Moro”, Pathological Anatomy, Bari, Italy

**Keywords:** Splenic abscesses, Crohn’s disease, Inflammatory bowel diseases

## Abstract

**Background:**

Splenic nodules are uncommon entities that occur rarely in the general population. Although an infectious etiology (primarily bacteria, followed by mycobacteria) is usually found, noninfectious diseases, including malignancies and autoimmune disorders, can also be involved. For instance, in course of inflammatory bowel diseases (IBDs), in particular Crohn’s Disease, aseptic splenic abscesses have been reported in patients with a long history of illness, or in those unresponsive to medical treatments, while are only anecdotally reported in the early phase of the disease. Hence, we presented the case of aseptic splenic nodules as a first manifestation of Crohn’s Disease.

**Case presentation:**

A 21-year-old woman with a silent medical history was admitted to the Emergency Department of our hospital complaining of fever of 38–39 °C (mainly in the evening) for the past 10 days and left flank abdominal pain, accompanied by sweating and fatigue. An abdominal computed tomography showed multiple splenic nodules of unknown origin. Because of the absence of clinical improvement after several antibiotic therapiesand a positron emission tomography (PET) with hypercaptation strictly localized to spleen, she underwent splenectomy, in suspicion of lymphoma. For persistence of symptoms after splenectomy, she underwent many instrumental examination, including a colonoscopy with bowel and intestinal biopsies that poses diagnosis of Crohn’s disease. A second PET confirmed this diagnosis showing this time also the gastrointestinal involvement.

**Conclusion:**

An unusual onset of Crohn’s disease with multiple splenic nodules is reported. This case suggests that in light of splenic nodules of unknown etiology attention should be paid to all possible diagnoses of aseptic abscesses, including IBDs (primarily Crohn’s Disease).

## Background

Splenic nodules are rare entities that occur at a low frequency in the general population (between 0.14 and 0.7%) [1], whose etiological definition can be quite troubling since they can be observed under several different conditions although they are more frequently caused by infectious diseases (Table 1). Aerobes (gram-positive cocci and gram-negative bacilli), anaerobes (mostly Bacteroides spp. and Clostridium spp.), and fungi (Candida spp. and Aspegillus spp.) are often involved indeed, causing either monomicrobial or polymicrobial splenic abscesses [2]. In particular, gram-negative bacteria and anaerobes should be suspected in case of intra-abdominal infections; in immunocompromised hosts, however, gram-negative bacteria, fungi and mycobacteria should be considered in the differential diagnosis even independently from the infectious site [1, 2]. In addition, cases of tubercular granulomas of the spleen or secondary splenic localization of atypical disseminated mycobacteriosis are repeatedly reported in the literature [3, 4].

**Table 1:** Main causes of splenic abscesses

Main causes of splenic abscesses	
Bacterial Infections	- Gram positive bacteria (Staphilococcus spp., Streptococcus spp., Clostridium spp., etc)
	- Gram negative bacteria (Enterobacteriacee, non fermenting Gram Negative bacteria, etc)
	- Rare organisms (Nocardia spp., Actynomices spp., etc)
Fungal Infections	- Candida spp., Aspergillus spp., Endemic Fungi, etc
Mycobacterial Infections	- M. tuberculosis spp., Atypical Mycobacteria spp.
Blood Cancers	- Lymphoma/Leukemia
Solid Cancers	- Metastasis
Auto-immune Disorders	- Reumatoid Arthritis
	- Systemic Lupus Erythematosus
	- Dermato/Polymyositis
	- Sarcoidosis
	- Systemic Vasculitis (Polyarteritis Nodosa, Granulomatosis with Polyangitis, etc)
	- Inflammatory Bowel Diseases
Genetic Diseases	- Common Variable Immunodeficiency
	- Rare Genetic Immunodeficiency Disorder

Many other noninfectious diseases can also be associated with aseptic splenic abscesses: lymphoma, leukemia, and metastastatic solid tumors are other important illnesses to investigate and consider for differential diagnosis [2]. Similarly, splenic involvement could occur in course of disseminated autoimmune disorders such as common variable immunodeficiency, inflammatory bowel diseases (IBDs) [5, 6], systemic lupus erythematosus [7], rheumatoid arthritis [8], or sarcoidosis [9]. However, in these cases, splenic abscesses emerge as late complications of disease in patients unresponsive to medical treatments, or with long history of illness. On the contrary, splenic nodules have been rarely observed in early phases of these autoimmune diseases. Thus, we presented an unexpected case of multiple splenic abscesses as an ascertained first manifestation of Crohn’s disease.

## Case presentation

**Fig. 1:**
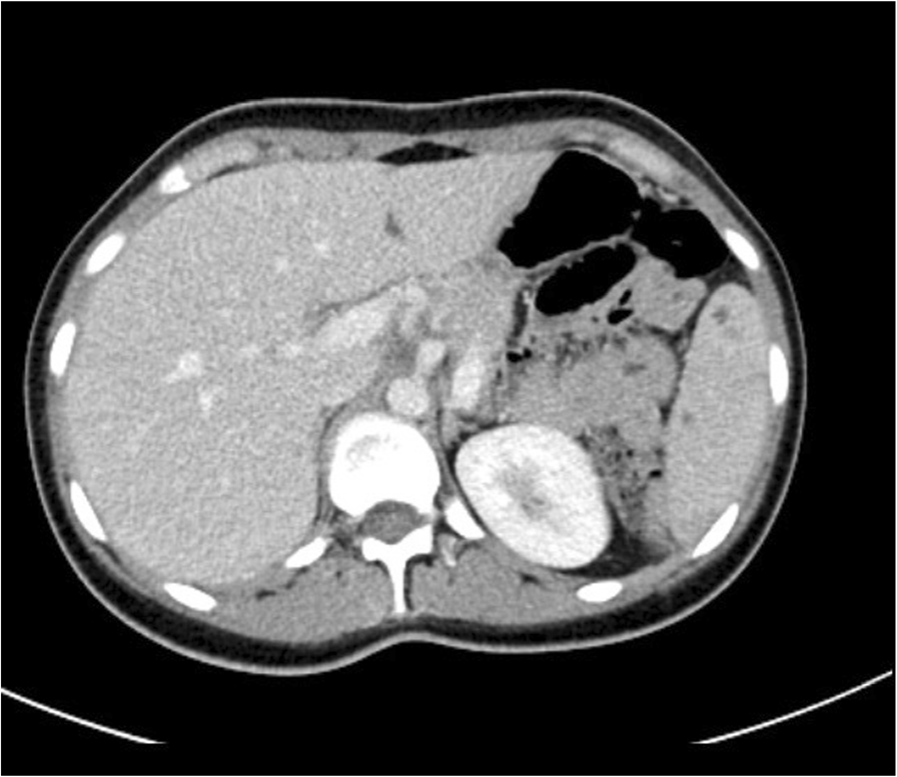
Abdominal CT scan. The CT scan shows multiple anechoic hypoechogenic nodules of the spleen, suggestive of abscesses or splenic lymphoma

A 21-year-old woman with a silent medical history was admitted to the Emergency Department of the University Hospital of Bari (Italy), complaining of a fever of 38–39 °C (mainly in the evening) for the past 10 days and left flank abdominal pain, accompanied by sweating and fatigue. She reported no weight loss, drug consumption, or traumatic events. Blood exams showed leukocytosis (16.62 × 10^3^/μL), elevated platelets (506 × 10^3^/μL), ESR (99 mm/h), CRP (131 mg/L), and PCT (0.10 ng/mL), while abdominal CT scan (Fig. 1) revealed celiac-mesenteric lymphoadenopathy, splenomegaly and the presence of multiple anechoic hypoechogenic nodules of unknown origin. Therefore, she was admitted to our Infectious Diseases Unit for further investigations.

Based on the most common etiologies, many laboratory investigations were done, beginning with microbiological exams: blood cultures, urine cultures, copro-cultures, Mantoux test and Quantiferon TB Gold, serologies for viruses (HIV, HCV, HBV, CMV, EBV, HSV 1/2, and VZV), Toxoplasma spp., Amoeba spp., Echinococcus spp., and Leishmania, and immunoglobulins and tumor markers dosage.

Because of her persistent fever and elevated inflammatory markers, the patient commenced empirical antibiotic therapy with Piperacillin/Tazobactam and Gentamicin.

However, all aforesaid investigations and both transthoracic echocardiography and abdominal ultrasonography were negative, while inflammatory markers remained elevated (CRP 120 mg/L, ESR 120 mm/h, CBC 12.23 × 10^3^/μL), and her fever slightly decreased although it did not resolve despite 10 days of antibiotics, which were therefore discontinued, due to ineffectiveness.

**Fig. 2:**
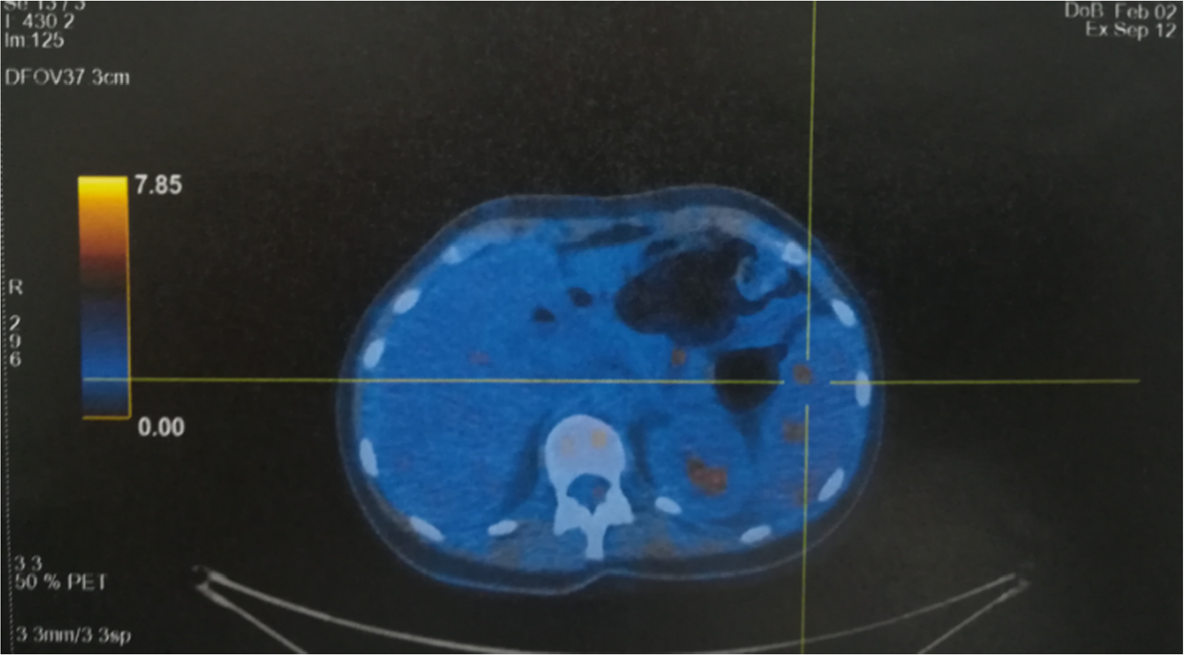
CT/PET Total Body CT/PET Total Body shows hypercaptation of 18F-fluorodeoxyglucose (FDG) in the spleen

Owing the high suspicion of splenic neoplastic localization, the patient underwent a PET (Fig. 2) that showed a pathologic hypercaptation strictly localized to splenic nodules and retroperitoneal lymph nodes, strengthening the diagnostic hypothesis of lymphoma. Therefore, after consultation with a hematology specialist and a general surgeon, the patient underwent splenectomy and then was discharged, to wait at home for the histological results.

**Fig. 3:**
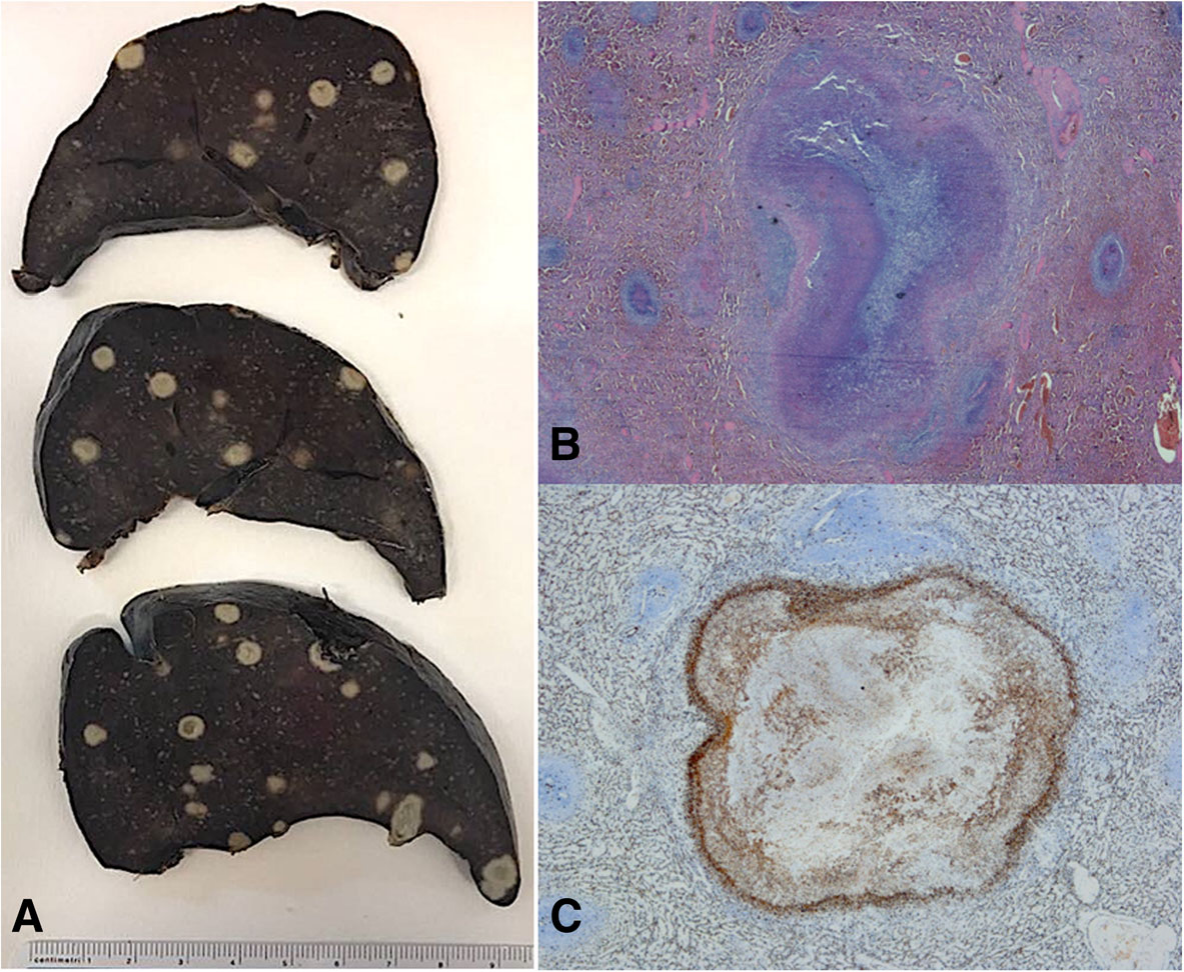
Splenic histology. Panel **a**: Macroscopic examination of the spleen. Panel **b**: Microscopic examination of a spleen nodule showing areas of necrosis surrounded by an organized epithelioid macrophage reaction (original magnification × 20). Panel **c**: Microscopic examination of a spleen nodule displaying CD68 immunoreactivity confirming the presence of activated monocyte/macrophage cells surrounding the necrosis area (original magnification × 20)

Macroscopic examination of the splenic cut surface showed many nonconfluent white nodules (Fig. 3, panel a). Histologically, spleen nodules showed numerous areas of necrosis (composed of neutrophils and cellular debris) surrounded by an organized epithelioid macrophage reaction (Fig. 3, panel b), displaying CD68 immunoreactivity (Fig. 3, panel c). An extensive immunohistochemical study excluded a primitive splenic lymphoma.

The PAS, PAS diastase, Ziehl-Neelsen, and Grocott stains demonstrated the absence of fungal and mycobacterium organisms, suggesting a bacterial necrotizing process. However, all microbiology investigations on the spleen samples were negative.

Three weeks after the splenectomy, the patient was readmitted with persistent mild grade fever (38 °C) and general malaise. Additional blood samples were collected to test for Brucella, Bartonella, Yersinia spp., Campylobacter spp., Coxiella, Trichinella spp., and Shigella spp., and stools samples were obtained to perform DNA PCR for Yersinia spp., Campylobacter spp., Salmonella spp., Shigella spp., *E. coli* (EIEC, STEC/VTEC), and *Clostridium difficile*. The laboratory investigations were completed by assessing neutrophil activity, Burst Test and Angiotensin Converting Enzyme dosage; anyway, all requested tests were negative.

Concurrently, a new antibiotic empiric cycle was started with Piperacillin/Tazobactam and Vacomycin. During hospitalization, the patient became afebrile, but leukocytosis (15.56 × 10^3^/uL) and inflammatory markers (ESR 120 mm/h, CRP 54 mg/L) remained elevated. An ex-juvantibus anti-mycobacterial therapy with Isoniazid, Rifampicin, Etambutol and Clarithromycin, in suspect of disseminated atypical mycobacteriosis, was then attempted.

**Fig. 4:**
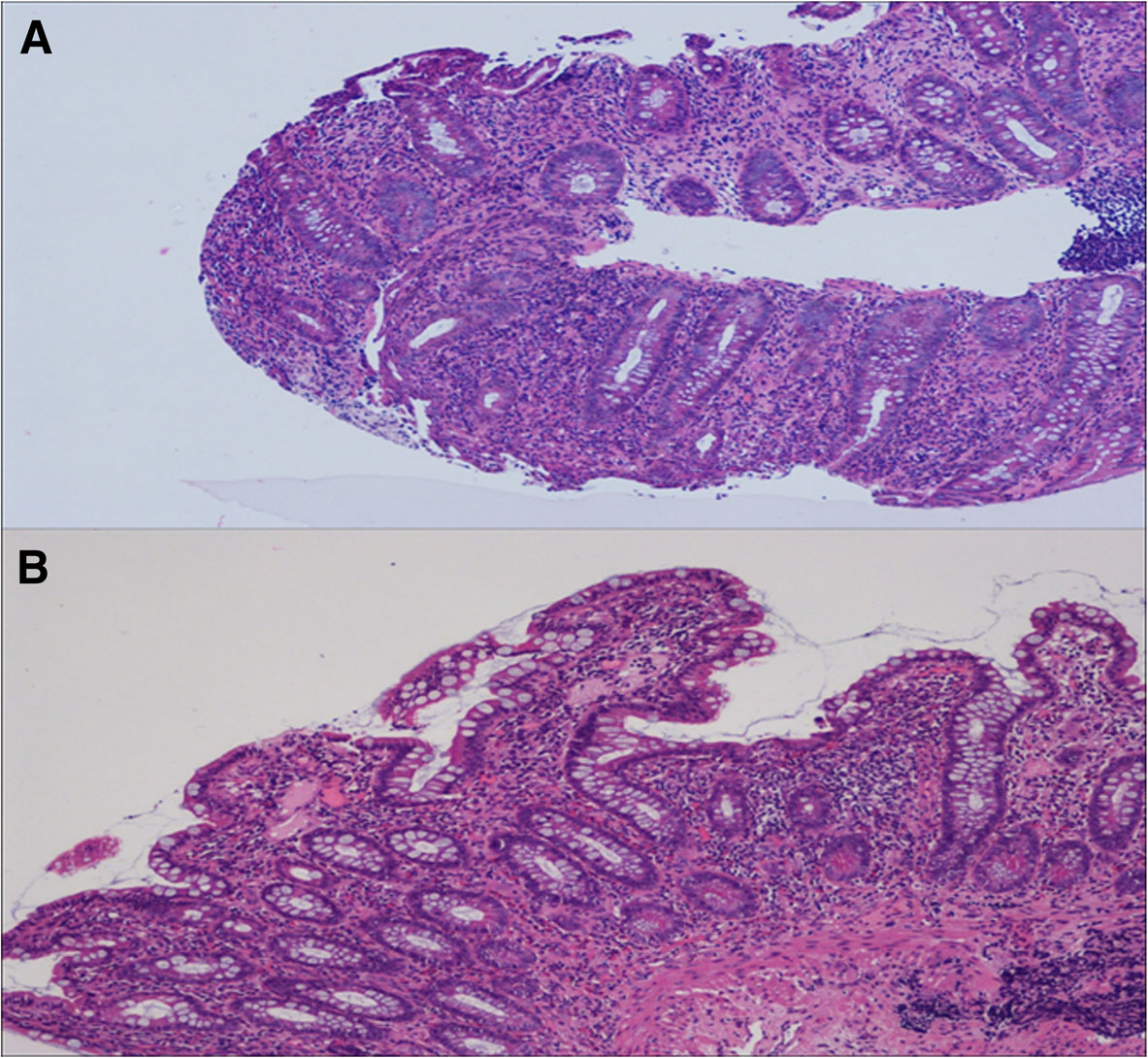
Histological examination of the gut. Microscopy of the descending colon (**a**) and ileum (**b**) showing chronic inflammatory infiltrate, glandular distortion and aphthous erosions, all highly suggestive of Crohn’s disease (hematoxilin and eosin, original magnification × 100)

Moreover, trans-esophageal echocardiography (TEE) and gastroscopy were performed, but the results were negative for both. Finally, the patient underwent a colonoscopy with evidence of multiple superficial mucosal ulcerations of the descending colon and rectal sparing. Histological examination revealed aphthous erosions characterized by focal surface epithelial necrosis associated with mixed chronic inflammatory infiltrate, foci of glandular distortion and the absence of granulomas, all of which are consistent with Crohn’s disease (Fig. 4). Concomitantly, we received the results of auto-antibodies assessment, performed during the previous hospitalization, that resulted negative, apart from positive ASCA IgG results.

**Fig. 5:**
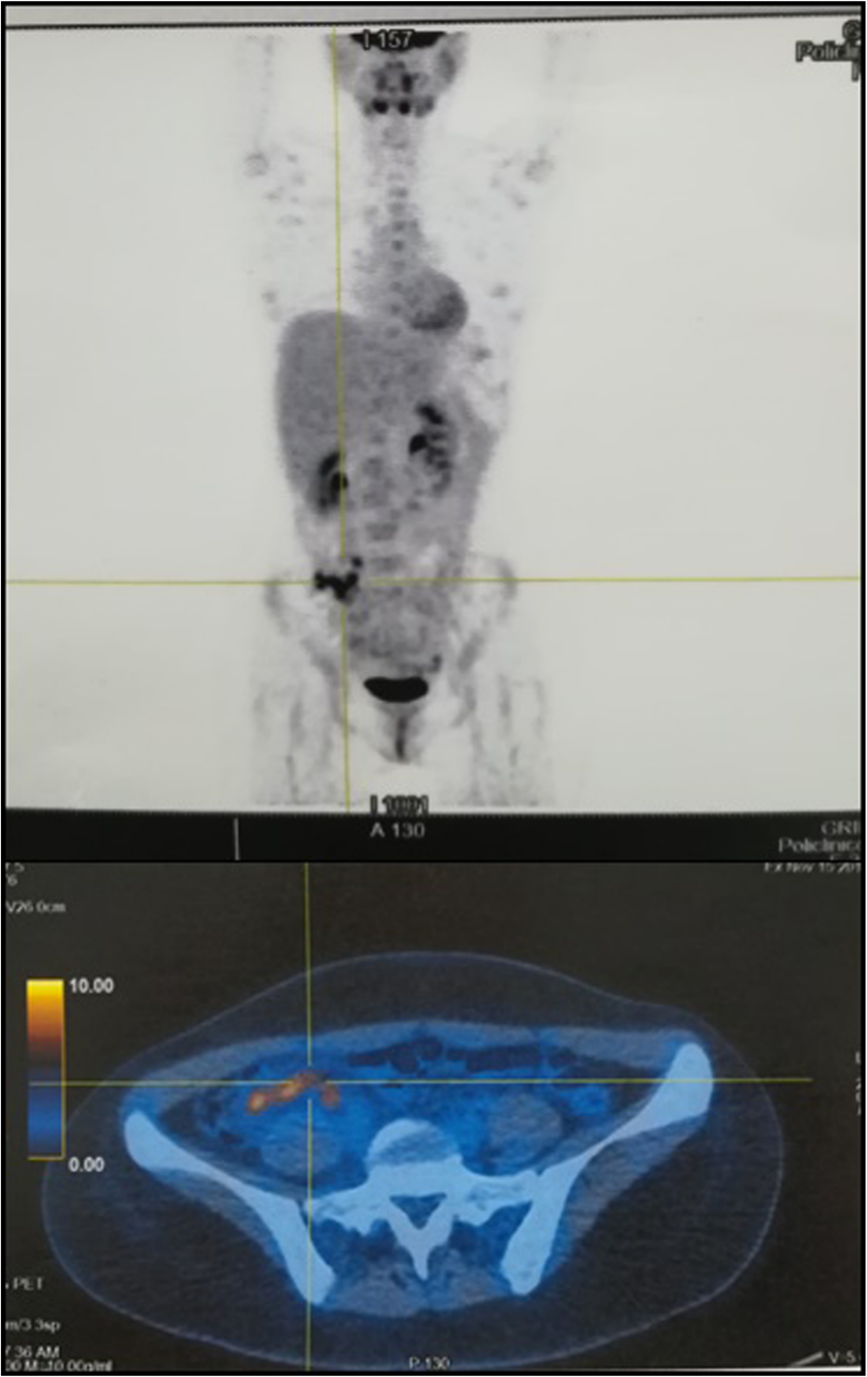
Second CT/PET Total Body. The second CT/PET shows ^18^F-DFG accumulation only in the descending colon

The patient also underwent a new PET displaying an unexpected, newly onset pathological capture in the lower gastrointestinal tract that was not evident in the previous PET (Fig. 5). Hence, after almost three months of investigations, a diagnosis of Crohn’s Disease was made on the basis of colon biopsies, positive ASCA IgG results and considering that splenic nodules are possible complications of Inflammatory Bowel Diseases (IBDs), even if this very rare complication generally occurs in the later stages of the disease.

The diagnosis was also made on the basis of anecdotical cases in the literature showing that splenic nodules can arise simultaneously with colonic manifestations or even precede gastrointestinal involvement.

In conjunction with the gastroenterology specialists, the anti-mycobaterial treatment was discontinued and she commenced corticosteroids and mesalazin. After one week, her blood tests showed a substantial reduction of inflammatory markers (CRP 17.6 mg/L and ESR 110 mm/h), and she was discharged at home in good clinical condition with a diagnosis of extraenteric manifestations of Crohn’s disease.

## Discussion and conclusions

Splenic abscesses are a rare and ambiguous clinical entity associated with a wide range of diseases. Differential diagnosis is often problematic, particularly if there are no other particular signs or symptoms that can direct the diagnostic workup. Given this wide variety of alternative possibilities, patients affected by splenic nodules of unknown origin often require long hospitalization, a deep anamnesis, and a wide range of laboratory tests, including the assessment of autoimmune diseases [6–9] and microbiological investigations to exclude infections caused by bacteria, fungi and mycobacteria [2–4]. Among autoimmune diseases, the onset of Crohn’s Disease as splenic abscesses is very rare. In fact, Crohn’s Disease may affect any part of the gastrointestinal tract from the mouth to the anus [10] but can also cause many different extra-enteric manifestations either at earlier or later disease phases. Among the wide range of manifestations, musculoskeletal and dermatologic systems are the most commonly affected, although the involvement of the hepato-pancreato-biliary system (e.g., primary sclerosing cholangitis) and the ocular, renal, and pulmonary systems have also been described [11, 12]. Albeit Crohn’s extra-intestinal aseptic abscesses are well known and often described, in a literature search of the PUBMED database, only three papers reporting single cases of splenic abscesses as a first manifestation of disease, before the onset of clinically evident gastrointestinal manifestations were found [13–15]. In addition, a fourth paper from the “French Study Group on Aseptic Abscesses” describes 21 cases of aseptic splenic abscesses associated with IBDs, including 7 cases arisen before an IBD diagnosis was formulated [5].

Notably, to the best of our knowledge, this is the first time in the medical literature that the onset of splenic abscesses prior to gastroenteric manifestation of Crohn’s Disease is demonstrated with PET. In fact, the execution of two PETs at a one-month interval allowed us to effectively demonstrate that the splenic involvement preceded the onset of intestinal lesions.

In conclusion, an unusual onset of Crohn’s disease with multiple splenic nodules is reported. This case documents Crohn’s disease as a rare cause of aspetic splenic abscess, that should be considered among possible differential diagnosis of this ambiguos clinical entity.

## Data Availability

Not applicable.

## Abbreviations

ASCA: anti-saccharomyces cerevisiae antibodies
CMV: Cytomegalovirus
CRP: C reactive protein
CT scan: Computed tomography scan
EBV: Epstein-Barr virus
EIEC: Entero-invasive *E. coli*
ESR: Erythrocyte sedimentation rate
HBV: Hepatitis B virus
HCV: Hepatitis C virus
HSV: Herpes virus
IBDs: Inflammatory bowel diseases
PAS: Periodic acid-schiff
PCT: Procalcitonin
PET: Positron emission tomography
STEC: Shiga toxin-producing *E. coli*
TB: Tuberculosis
TEE: Trans-esophageal echocardiography
VTEC: Vero-toxin E.coli
VZV: Varicella-Zoster virus
